# Suit for Damages

**Published:** 1901-09

**Authors:** 


### SUIT FOR DAMAGES.

The attorneys selected by the committee from the Atlanta Society of Medicine have filed an answer to the suit brought against certain members of it in June by one E. S. E. Bryan. Bryan alleges in his suit that by direction of the Atlanta Medical Society his name was presented to the grand jury in June, 1900, as an illegal practitioner, and it is for that he seeks damages.

The answer sets forth that in the spring of 1900, a movement was inaugurated against illegal practitioners of medicine, and that a committee was appointed to investigate the matter. The plea says that this committee "did undertake in good faith and without malice to ascertain who were illegal practitioners, and to that end instituted a diligent search through the recognized city directories of Atlanta, through investigating signs and names upon office doors, advertisements, and the like, to ascertain who were holding themselves out as practicing medicine, and when a list of the persons so holding themselves out had been obtained, the committee then, in good faith and without malice, compared these names with the names of those physicians who were registered according to law."

It is alleged that the plaintiff's name was found in one of the recognized city directories as a physician, and that, according to the information the defendant had, he held himself out as practicing medicine in the county of Fulton. It was further alleged that the plaintiff had not registered in Fulton county, and that he held himself out as a physician in this county, and had an office in the city from November, 1900, to January, 1901. So far from any of the Society having any malice against the plaintiff, it is said in the answer that none of them had ever known or heard of him personally.

The answer also denies that the Society ever prosecuted the plaintiff and sets up that none of them ever appeared before the grand jury, but states that the list of names was merely given a member of the grand jury for that body to act upon as it saw fit.

The answer further states that while the plaintiff claims to practice medicine in DeKalb county, it has been discovered that he never registered there until the year 1898, and that an execution for unpaid professional taxes for that year is now outstanding against him.

